# Conformational Entropy as a Potential Liability of Computationally Designed Antibodies

**DOI:** 10.3390/biom12050718

**Published:** 2022-05-18

**Authors:** Thomas Löhr, Pietro Sormanni, Michele Vendruscolo

**Affiliations:** Department of Chemistry, University of Cambridge, Cambridge CB2 1EW, UK; tl465@cam.ac.uk (T.L.); ps589@cam.ac.uk (P.S.)

**Keywords:** antibody design, antibody engineering, protein design, metadynamics, molecular dynamics

## Abstract

In silico antibody discovery is emerging as a viable alternative to traditional in vivo and in vitro approaches. Many challenges, however, remain open to enabling the properties of designed antibodies to match those produced by the immune system. A major question concerns the structural features of computer-designed complementarity determining regions (CDRs), including the role of conformational entropy in determining the stability and binding affinity of the designed antibodies. To address this problem, we used enhanced-sampling molecular dynamics simulations to compare the free energy landscapes of single-domain antibodies (sdAbs) designed using structure-based (DesAb-HSA-D3) and sequence-based approaches (DesAbO), with that of a nanobody derived from llama immunization (Nb10). Our results indicate that the CDR3 of DesAbO is more conformationally heterogeneous than those of both DesAb-HSA-D3 and Nb10, and the CDR3 of DesAb-HSA-D3 is slightly more dynamic than that of Nb10, which is the original scaffold used for the design of DesAb-HSA-D3. These differences underline the challenges in the rational design of antibodies by revealing the presence of conformational substates likely to have different binding properties and to generate a high entropic cost upon binding.

## 1. Introduction

Antibodies have become essential tools in the fields of biological chemistry, medical diagnostics and therapeutics [1,2,3]. The technologies available to discover novel antibodies for a target of interest can be grouped into three broad categories. In vivo approaches utilize the immune system for raising antibodies against antigens of interest, in particular using transgenic animals to generate human antibodies [4,5] or the screening of B-cells isolated from patients [6]. In vitro techniques rely on the screening, for example, by phage display [7], of laboratory-constructed libraries to identify antibodies binding the desired target. We note, however, that the biophysical properties of in vitro-isolated antibodies are often inferior to those of antibodies obtained with in vivo methods [8,9,10,11,12,13,14].

*In silico* approaches to antibody and antibody-mimic design have recently started to provide an attractive alternative [9,15,16,17,18,19] and circumvent some of the limitations of laboratory-based approaches. Moving the costly and time-consuming work of isolating antibody sequences with desired characteristics to in silico can significantly accelerate the development time, and allow a more efficient search of sequence space. Moreover, computational approaches readily enable the targeting of predetermined epitopes of choice, which remains a challenge for laboratory-based methods [15,19].

We recently developed one such method, with the general idea of enabling the identification of peptides complementary to chosen epitopes on the target antigens [20]. These peptides can then be grafted onto a suitable antibody scaffold such as CDRs. In this approach, the complementarity is designed by mining the Protein Data Bank [21] for β-strand conformations, and by identifying suitable fragments paired with parts of an epitope sequence. By cascading along the sequence and identifying further fragments, the complementary peptide sequence can be constructed. We note that this method does not require the structure of the target epitope, but only its sequence and, therefore, we consider it a sequence-based approach. This approach has been successfully used to design single-domain antibodies (sdAbs) targeting the elusive and mostly disordered oligomers of the amyloid-β peptide found in Alzheimer’s disease [22,23]. Since this rational design method is limited to targeting unstructured epitopes ideally within amyloidogenic antigens, we recently extended this approach to target any structured epitopes on protein surfaces [24]. This approach also relies on fragment-based design but, rather than looking at β-strands only, it looks more generally at any fragment whose backbone structure is compatible with that of an antibody CDR. Since this approach is structure-based, it can also predict a model of the designed CDR bound to its epitope, for which a structure or an accurate model is required. This approach was experimentally validated by designing six sdAbs targeting different epitopes on three antigens [24].

Despite these advances, the structural and dynamical features of designed sdAbs are still largely unknown. Gaining a deeper insight into the behavior of the CDRs in solution could enable further improvements in the computational design stage, yielding higher affinities and improved biophysical properties. In particular, it is unknown what role the conformational entropy plays in the ability of the sdAbs to bind their targets with high affinity.

Previous molecular simulation studies suggest diverse kinetic and thermodynamic behavior in sdAbs from various sources [25], but did not investigate in silico designs. To better understand the dynamics of the CDRs, we performed molecular dynamics simulations with enhanced sampling of two designed sdAbs and one sdAb isolated from a camelid immune system. More specifically, we investigated the conformational dynamics of DesAbO [23], which targets amyloid-β oligomers and was generated using the sequence-based cascade method outlined above [20]. Similarly, we performed simulations of DesAb-HSA-D3, which was designed to target human serum albumin (HSA) using the aforementioned structure-based strategy [24]. Knowledge of the conformational flexibility could inform further optimizations to these in silico design techniques to further improve binding affinities.

Besides the different antigens, the key difference between these two designed sdAbs is in the way in which the designed CDRs are grafted onto the sdAb scaffold. The CDR of DesAbO was designed with a sequence-based approach, and the designed sequence was grafted in the CDR3 of an sdAb scaffold known to be highly tolerant to CDR3 replacement [20]. By contrast, DesAb-HSA-D3 was obtained with a structure-based design strategy. Models of CDR1 and CDR3 fragments bound to HSA were obtained and then structurally matched to an sdAb scaffold whose original CDRs were structurally compatible with the designed ones in their bound conformation, where the structural compatibility was defined based on backbone RMSD and lack of side-chain clashes [24]. Therefore, DesAbO has only a rationally designed CDR3, while DesAb-HSA-D3 has both designed CDR1 and CDR3, which were grafted on a structurally compatible scaffold. For comparison, we also ran simulations of the unmodified scaffold, called Nb10, used to generate DesAb-HSA-D3, which was developed using a llama immunization technique [26]. These sdAbs thus represent examples of different design approaches, with potentially different conformational properties (Table 1).

## 2. Materials and Methods

### 2.1. Simulation Details

All simulations were performed using GROMACS 2019.3 [27] and a development version of PLUMED 2.6 [28,29] (git commit 8859093). We chose CHARMM36m [30] as the force field, together with the TIP3P [31] water model. Starting conformations for DesAbO and DesAb-HSA-D3 were created using MODELLER [32], Nb10 was retrieved from the PDB structure 4DKA. The structures were solvated in a rhombic dodecahedron box with a volume of 214 nm${}^{3}$ (DesAb-HSA-D3: 197 nm${}^{3}$, Nb10: 227 nm${}^{3}$) using 6726 (DesAb-HSA-D3: 6103, Nb10: 6779) water molecules. Each system was energy minimized using the steepest descent algorithm to a target force of 1000 kJ/(mol/nm) and equilibrated over a period of 500 ps in the NVT ensemble with the Bussi thermostat [33], and over 5 ns in the NPT ensemble using Berendsen pressure coupling [34], while applying a position restraint on all heavy atoms, at a temperature of 300 K. The systems were then each simulated at 400 K for 5 ns in the NVT ensemble and 32 new starting structures were then sampled from the respective trajectories at random, to produce a set of diverse CDR conformations. Each conformation was then equilibrated using the same procedure outlined above, at a temperature of 300 K. Production simulations were performed at 300 K in the NPT ensemble using Parrinello–Rahman pressure coupling [35] with a time step of 2 fs. Constraints were applied using the LINCS algorithm [36] with a matrix expansion on the order of 4 and 1 iteration per time step. Modeling of electrostatic interactions was performed using the particle mesh Ewald [37] approach with a cut-off for short-range interactions at 1.2 nm. All simulations were performed using parallel-bias metadynamics [38], using the well-tempered [39] and multiple-walkers [40] protocols with 32 replicas (see Supplementary Information for details).

### 2.2. Analysis

**Figure 1:**
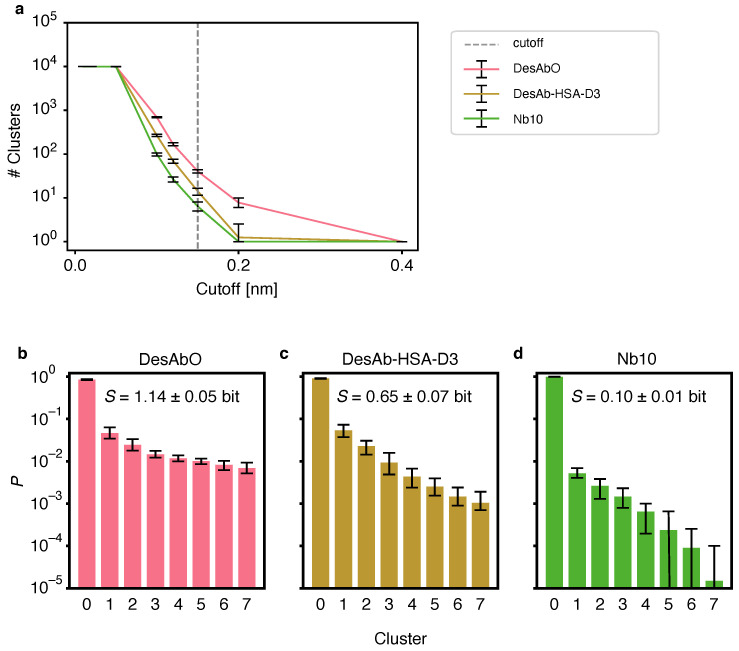
Comparison of the clustering of the conformational space of the CDRs of the 3 sdAbs studied in this work using the GROMOS [42] algorithm. (**a**) Number of clusters for varying cut-off values for all conformational ensembles. (**b**–**d**) Populations for the top 8 clusters for DesAbO (**b**), DesAb-HSA-D3 (**c**) and Nb10 (**d**); the mean information entropy (S) over the normalized populations is indicated. Error bars indicate the 95th percentile of the bootstrap sample-of-the-mean over all 20 samples consisting of 10,000 frames sampled from the ensemble based on the metadynamics weights.

The individual replica trajectories were concatenated and the time-independent bias calculated using PLUMED *driver* as described in [41]. The statistical weight for each frame *i* was calculated as $w_{i}=\exp\left(\frac{V_{\mathrm{PB}}}{k_{B}T}\right){\left[\sum_{i}\exp\left(\frac{V_{\mathrm{PB}}}{k_{B}T}\right)\right]}^{-1}$, where $V_{\mathrm{PB}}$ is the parallel-bias metadynamics potential, $k_{B}$ is the Boltzmann constant and *T* is the temperature [38]. All observables were calculated as weighted ensemble averages. Convergence was assessed by clustering 20 bootstrap samples of each trajectory and comparing the populations of each cluster in the case of the first and second halves of the respective simulations (Figure S1). We chose the GROMOS clustering algorithm [42] based on Cα RMSDs, as implemented in GROMACS 2019.3 with a cut-off of 0.15 nm based on the evaluation of several different values (Figure 1C). Convergence was assessed by clustering the whole trajectory using the same method, discarding the first 10% of frames and comparing the cluster populations between the remaining first and second halves of the simulation (Figure S1). We additionally performed clustering using a hierarchical algorithm with average linkage, using cut-offs between 0.01 nm and 0.4 nm, as implemented in scikit-learn [43]. Contacts between groups were defined as any inter-residue heavy-atom distance below 0.45 nm. Scaffold and CDR were defined as detailed in Table 1 and Table 2. Information entropies *S* over clusters were calculated using the relative population of each cluster $p_{i}$ as $S=-\sum_{i}p_{i}\log p_{i}$ as described in [44]. Dihedral entropies were calculated using normalized 2D histograms of 100 bins for all ϕ and ψ backbone dihedrals as $S=-\sum_{ij}p_{ij}\log p_{ij}$ where $p_{ij}$ represents each bin.

## 3. Results

### 3.1. Metadynamics Simulations of the sdAbs

We performed all-atom, explicit water, parallel-bias metadynamics [38] simulations of DesAbO, designed using a sequence-based method [20], DesAb-HSA-D3, built using a structure-based approach [24], and Nb10, developed using llama immunization (Table 2). After 9.3 µs, all simulations were found to be largely converged for cluster populations larger than 10 (out of 10,000) frames (Figure S1).

### 3.2. The Designed Antibodies Exhibit High Conformational Entropy

To evaluate conformational heterogeneity of the CDRs, we performed a clustering analysis using the GROMOS algorithm [42] (Figure 1). We evaluated several different cut-off values for the Cα root mean square deviation (RMSD) and found that the number of identified clusters varied strongly between the conformational ensembles (Figure 1b). To facilitate the comparison between clusters we chose a cut-off of 0.15 nm for all systems. The per-cluster population decays fastest for the Nb10 ensemble (Figure 1a), indicating lower structural heterogeneity and a more compact conformational landscape in this antibody. The second-fastest decay is found for DesAb-HSA-D3, while DesAbO features a more heterogeneous distribution. Based on the normalized cluster populations we calculated the information entropy over all clusters (Figure 1a), again indicating highest conformational flexibility in the DesAbO ensemble, followed by the ensembles of DesAb-HSA-D3 and lastly Nb10. We also note that, while the results in Figure 1b–d depend on the chosen cluster cut-off value, the trends in Figure 1a confirm that the resulting conformational flexibility ranking is robust with respect to this choice. To circumvent possible bias by the choice of the clustering algorithm, we repeated this process using a hierarchical approach (Figure S2). We again find a consistent ranking in terms of cluster population decay, albeit with a higher number of total clusters with high similarities among each other. The relative increase in flexibility is limited to the CDR3, with the CDR1 exhibiting coil-like behavior, and the CDR2 showing a stable fold across all ensembles (Figure S3). We note, however, that these two regions were not subject to a metadynamics bias potential, and thus may not have been sampled exhaustively. We further looked at the Ramachandran entropy for each peptide bond for all three sdAbs as a further metric for loop flexibility (Figure S4), analogous to the approach described in [45]. We find similar entropy patterns over all three ensembles, with the sequence- and structure-based designs (DesAbO and DesAb-HSA-D3) showing the highest flexibility in the CDR3 loop. On the other hand, both DesAb-HSA-D3 and Nb10 exhibit higher entropies in the CDR1 region.

### 3.3. Conformational Flexibility in the Complementarity-Determining Regions

**Figure 2:**
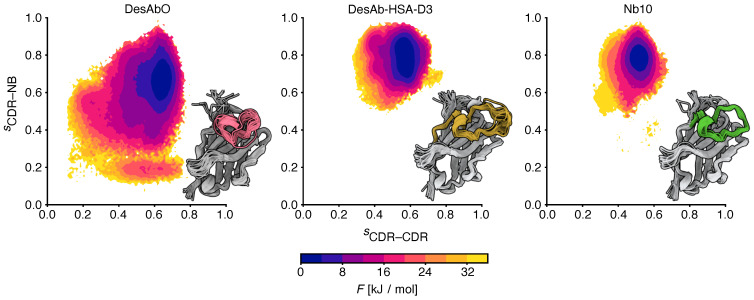
Comparison of the free energy landscapes of the 3 sdAbs studied in this work. The x and y axes represent the relative proportion of intra-CDR3 (CDR–CDR) and CDR3–scaffold (CDR–NB) contacts, respectively. Twenty randomly sampled structures are shown from each ensemble, with the CDR3 loop colored.

**Figure 3:**
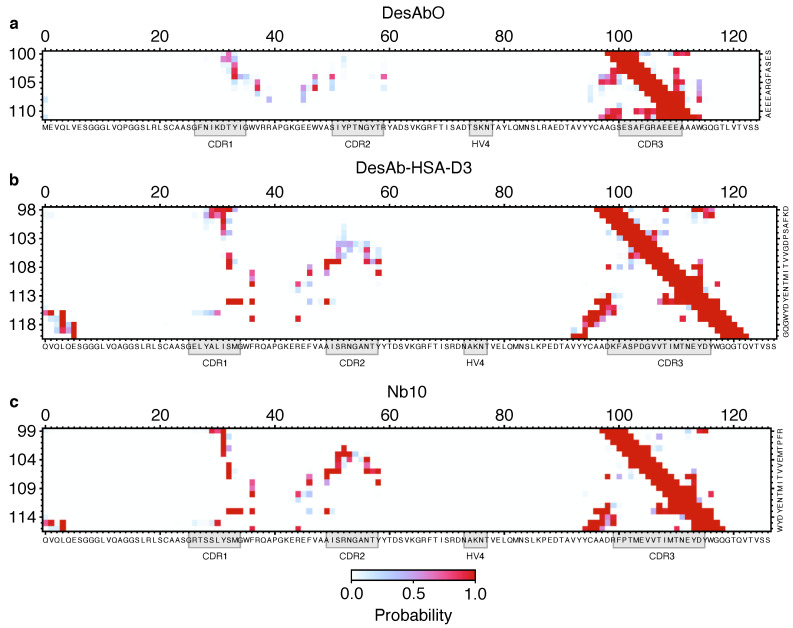
Comparison of the structural properties of the 3 sdAbs studied in this work. Contact maps for DesAbO (**a**), DesAb-HSA-D3 (**b**) and Nb10 (**c**) for CDR–scaffold contacts, indicating the probability of forming a contact between two residues.

To better understand the conformational properties of the sdAbs obtained by sequence-based design (DesAbO), structure-based design (DesAb-HSA-D3) and in vivo immunization (Nb10), we projected the free energy on to collective variables encoding the number of intra-CDR3 and CDR3–scaffold contacts (Figure 2). While the DesAb-HSA-D3 ensemble is relatively compact and maintains a large number of CDR–scaffold contacts, the DesAbO ensemble shows far fewer contacts not only with the scaffold, but also internally within the CDR3. Even in the lowest free energy state, the DesAbO system forms relatively fewer contacts with the scaffold than DesAb-HSA-D3 and its parent scaffold Nb10. The origin of this higher flexibility in the sequence-based design can be seen, for example, by the absence of contacts with residues 45–60 of the scaffold (Figure 3). We further see a reduction in the contact probability between the first part of the CDR and residues 30–35 in the scaffold, thus allowing for higher conformational heterogeneity. We note that while it may appear that DesAbO occupies two distinct minima, the population of this higher energy state is extremely low (0.1%), despite being sampled exhaustively by multiple replicas. Compared with Nb10, both in silico designs lack strong contacts of the CDR3 with residues 50–55 in the scaffold (Figure 3c), this is also reflected in a further narrowing of the free energy landscape (Figure 2). While only the CDR3 loop was biased during the simulation to enhance sampling, we also evaluated the flexibility and contacts of the other CDRs and the scaffold in general. While contacts formed by the CDR1 are similar across all three sdAbs (Figure S5), the CDR2 in DesAbO exhibits very few contacts with the CDR3 (Figure S6A). We further investigated the adjacent HV4 loop (Table 2), which has previously been shown to have a significant effect on antigen-binding affinity and often exhibits interactions with the CDR2 and CDR3 [46,47]. Contact maps (Figure S7) and free energy surfaces (Figure S8) show increased contact formation for the D3 scaffold (DesAb-HSA-D3 and Nb10) compared to DesAbO. Nb10 notably exhibits two-state behavior, however, we would like to note that this region of the protein was not explicitly biased using metadynamics. In terms of overall flexibility, the root mean square fluctuation across residues is significantly increased in DesAbO (Figure S9) compared to both DesAb-HSA-D3 and Nb10. The CDR2 flexibility is on par with CDR1 and CDR3 in DesAbO, but lower in DesAb-HSA-D3 and Nb10.

## 4. Discussion

The molecular dynamics simulations that we reported in this work indicate clear structural differences between all three antibodies at a conformational ensemble level. Notably, despite the increased length of the CDR3 of DesAb-HSA-D3, theoretically allowing more flexibility, this CDR is in fact more structured, with stronger CDR–scaffold contacts. As the design process for this sdAb specifically optimizes for structural stability, this is not surprising. Nb10 and DesAb-HSA-D3 share the same sdAb scaffold and exhibit many of the same CDR contacts (Figure 3), although those of Nb10 have a higher probability, consequently exhibiting a free energy minimum characteristic of a more structured conformation. In contrast, the rationally designed DesAbO is missing many interactions between the first half of the CDR3 and residues 50 to 60 in the scaffold. The lack of these particular contacts may be sufficient to decrease the rigidity of the CDR, and potentially impact binding affinities. Previous studies have emphasized the positive effect on binding affinity of more rigid CDRs [48]. On the other hand, higher conformational entropy in the loop might be beneficial in binding more disordered targets such as misfolded protein oligomers. In that case, the necessary structural rearrangements to form a β-sheet structure with the epitope may present a significant entropic barrier. Verifying these behaviors is difficult, as the structures of these oligomers remain elusive [49].

These results underscore the importance of taking into account the effects of the CDR–scaffold interactions in the sequence-based antibody design procedure. We thus suggest that an avenue to further optimize the binding affinity of computationally designed antibodies may be to tune these interactions. For example, mutations may be designed to make the formation of β-sheets easier, by selecting the residues of the CDR appropriately, or by engineering its stems to create anchor points on either side of the CDR to force a particular arrangement. However, the general role of rigidity in antibody–antigen binding remains unclear, with some results indicating only a slight reduction in dynamics in antibodies produced through affinity maturation compared to naïve antibodies [50], and others suggesting an increase in rigidity together with an increase in affinity [51]. Other studies hint at the role of water in the binding process and the entropically favorable formation of salt bridges [52,53]. The CDRs of a diverse range of antibodies have been studied using molecular dynamics simulations and Markov state models [25], revealing multiple CDR substates with microsecond-timescale transitions, and indicating potentially beneficial effects of conformational heterogeneity.

Taken together, the results that we reported indicate that the conformational entropy is a property that needs to be specifically optimized in the design of antibodies using computational methods.

## Figures and Tables

**Table 1:** Amino acid sequences of the 3 sdAbs studied in this work.

sdAb	Sequence
DesAbO	MEVQLVESGGGLVQPGGSLRLSCAASGFNIKDTYIGWVRRAPGKGEEWVASIYPTNGYTRYADSV
	KGRFTISADTSKNTAYLQMNSLRAEDTAVYYCAAGSESAFGRAEEEAAAWGQGTLVTVSS
DesAb-HSA-D3	QVQLQESGGGLVQAGGSLRLSCAASGELYALISMGWFRQAPGKEREFVAAISRNGANTYYTDSVK
	GRFTISRDNAKNTVELQMNSLKPEDTAVYYCAADKFASPDGVVTIMTNEYDYWGQGTQVTVSS
Nb10	QVQLQESGGGLVQAGGSLRLSCAASGRTSSLYSMGWFRQAPGKEREFVAAISRNGANTYYTDSVK
	GRFTISRDNAKNTVELQMNSLKPEDTAVYYCAADRFPTMEVVTIMTNEYDYWGQGTQVTVSS

**Table 2:** Definition of the complementarity-determining regions of the 3 sdAbs studied in this work.

sdAb	CDR1	CDR2	CDR3	HV4
DesAbO	GFNIKDTYIG (27–36)	SIYPTNGYTR (51–60)	SESAFGRAEEEA (101–113)	TSKNT (75–80)
DesAb-HSA-D3	GELYALISMG (27–36)	AISRNGANTY (51–60)	DKFASPDGVVTIMTNEYDY (99–118)	NAKNT (74–79)
Nb10	GRTSSLYSMG (27–36)	AISRNGANTY (51–60)	RFPTMEVVTIMTNEYDYW (99–118)	NAKNT (74–79)

## Data Availability

Analysis code and PLUMED input files are available from https://github.com/vendruscolo-lab/sdab-entropy, accessed on 7 May 2022.

## Supplementary Materials

The following supporting information can be downloaded at: https://www.mdpi.com/article/10.3390/biom12050718/s1, Figure S1: Simulation convergence for both sdAbs.; Figure S2: Agglomerative clustering algorithm with different cut-off values.; Figure S3: Comparison of the contact maps of CDR1 and CDR2.; Figure S4: Ramachandran entropy per residue.; Figure S5: Comparison of the contact maps of CDR1 with the scaffold.; Figure S6: Comparison of the contact maps of CDR2 with the scaffold.; Figure S7: Comparison of the contact maps of the HV4 loop with the scaffold.; Figure S8: Free energy surfaces of the HV4 loop region.; Figure S9: RMSF per residue. Reference [54] is cited in the supplementary materials.
