# Supplementary material for: Conformational Entropy as a Potential Liability of Computationally Designed Antibodies
Source: Biomolecules. 2022 May 18;12(5):718. doi: 10.3390/biom12050718 (PMC9138470; doi:10.3390/biom12050718)
Supplement: Supplementary file 1 [file biomolecules-12-00718-s001.zip › biomolecules-1646856-supplementary.pdf]

Supplementary Materials: Comparison of the conformational entropy of designed and natural nanobodies

Thomas Löhr [1], Pietro Sormanni [1] and Michele Vendruscolo [1,*]

1. Metadynamics details

Chosen collective variables were the following:

1. The sum of all backbone dihedral angles θ_i (ALPHABETA action in PLUMED) in the CDR:
$$s = \frac{1}{2}\sum_i \left[1 + \cos(\theta_i - \theta_i^{\text{ref}})\right] \tag{1}$$
where $\theta_i^{\text{ref}} = \pi$. The width was chosen as 1.5 rad.
2. The sum of side chain χ_1 dihedral angles in the CDR, also using Eq. 1. The width was chosen as 0.5 rad.
3. The root-mean-square deviation (RMSD) of consecutive residues in the CDR to an ideal α-helix (ALPHARMSD in PLUMED)[1]:
$$s = \sum_i \frac{1 - \left(\frac{r_i - d_0}{r_0}\right)^n}{1 - \left(\frac{r_i - d_0}{r_0}\right)^m} \tag{2}$$
where r_i is the RMSD of a six-residue fragment to an ideal α-helix, $r_0 = 0.08$ nm is the reference distance for the switching function, and $n = 8$ and $m = 12$ are parameters for the switching function. The width was chosen as 2.0.
4. The root-mean-square deviation of consecutive residues in the CDR to an antiparallel β-sheet (ANTIBETARMSD in PLUMED), using Eq. 2 with the same switching function parameters[1]. The width was chosen as 1.5.

All collective variables were weighted equally. Gaussians were deposited every 500 steps (1 ps) using an initial height of 1.2 kJ/mol and a bias-factor of 24. Systems were run for 293 ns (DesAb-HSA-D3: 292 ns, Nb10: 295 ns) per replica, totalling 9.3 µs (DesAb-HSA-D3: 9.3 µs, Nb10: 9.4 µs) of cumulative sampling. The sampling quality should be largely independent of the number of replicas, see [2].

References

1. Pietrucci, F.; Laio, A. A Collective Variable for the Efficient Exploration of Protein Beta-Sheet Structures: Application to SH3 and GB1. *5*, 2197–2201. doi:10.1021/ct900202f.
2. Raiteri, P.; Laio, A.; Gervasio, F.L.; Micheletti, C.; Parrinello, M. Efficient Reconstruction of Complex Free Energy Landscapes by Multiple Walkers Metadynamics. *110*, 3533–3539. doi:10.1021/jp054359r.

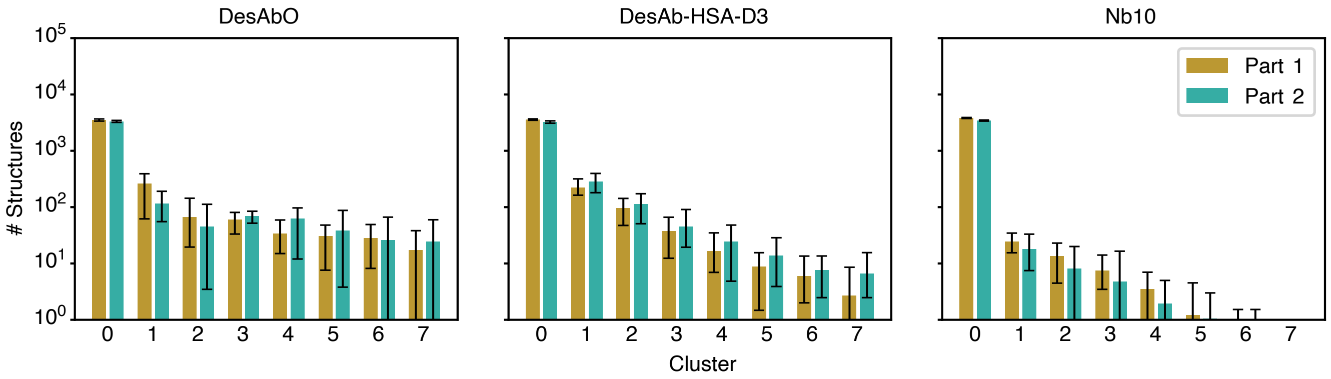

Figure S1. Convergence of the simulations for the DesAbO (**a**), DesAb-HSA-D3 **b** and Nb10 (**c**). After discarding the first 20% of the frames, simulations were split into equal blocks (Part 1 and Part 2) and the cluster populations compared. Error bars indicate the 95th percentile of the bootstrap sample-of-the-mean over all 20 samples consisting of 10000 frames sampled from each block based on the metadynamics weights.

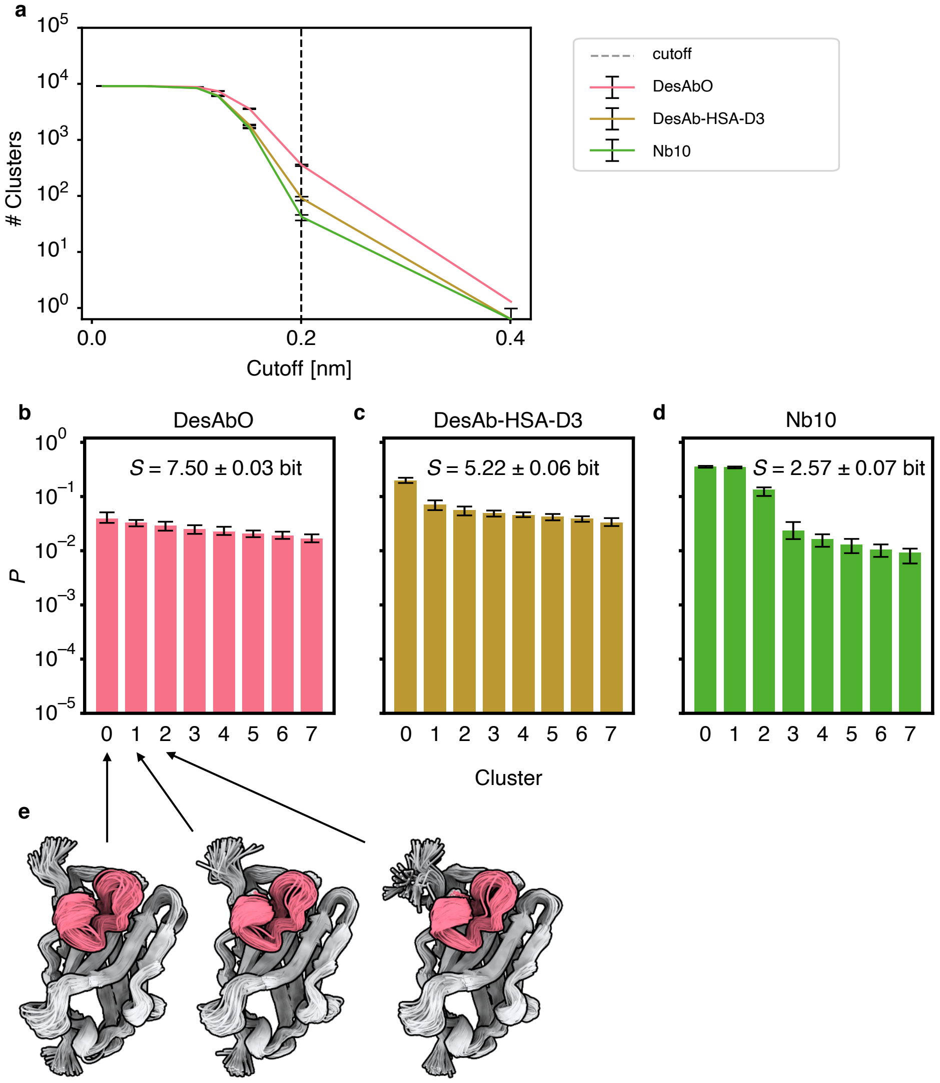

Figure S2. Comparison of the clustering of the conformational space of the CDRs of the 3 sdAbs studied in this work using agglomerative clustering. (**a**) Number of clusters for varying cut-off values for all conformational ensembles.(**b-d**) Populations for the top 8 clusters for DesAbO (**b**), DesAb-HSA-D3 (**c**), and Nb10 (**d**) with representative structures shown for DesAbO (**e**); the mean information entropy (S) over the normalised populations is indicated. Error bars indicate the 95th percentile of the bootstrap sample-of-the-mean over all 20 samples consisting of 10000 frames sampled from the ensemble based on the metadynamics weights.

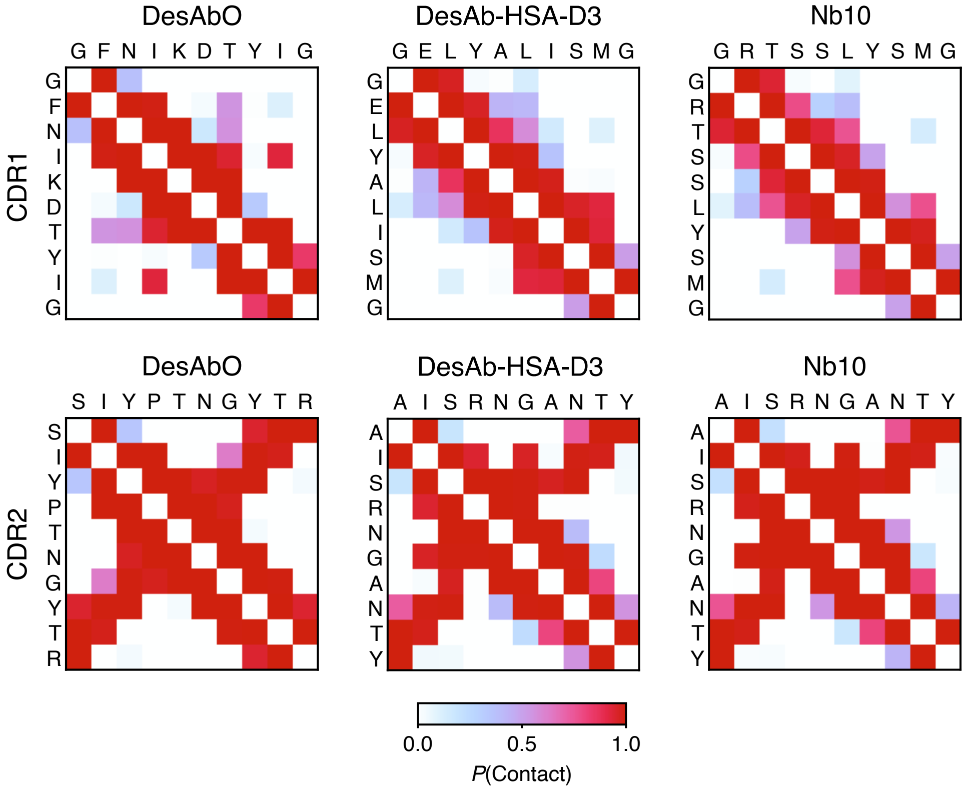

Figure S3. Comparison of the contact maps of intra-CDR contacts for CDR1 (top) and CDR2 (bottom) for all 3 conformational ensembles.

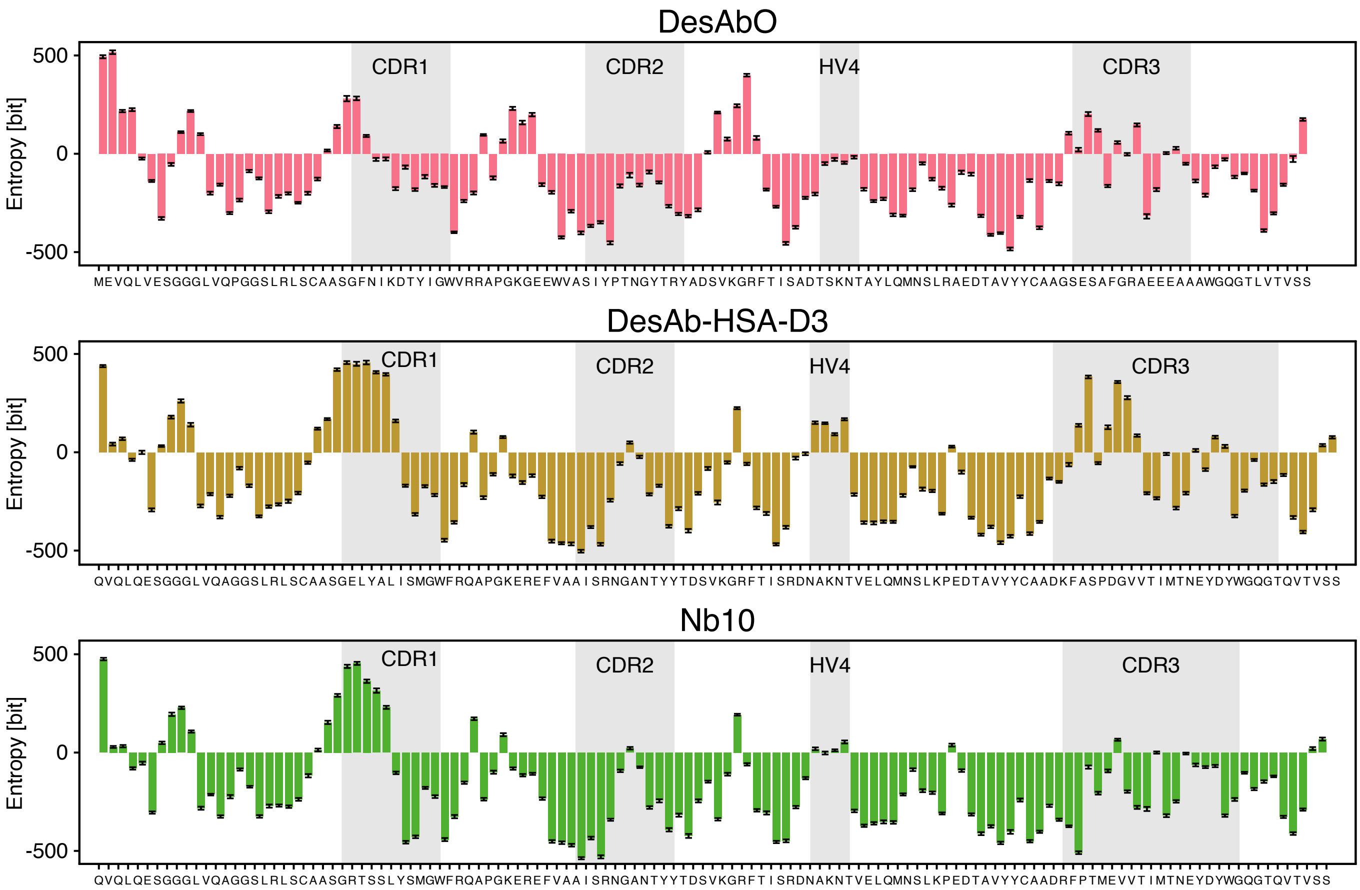

Figure S4. Ramachandran entropy per peptide bond, calculated as the information entropy over the Ramachandran distribution. Error bars indicate the 95th percentile of the bootstrap sample-of-the-mean over all 20 samples consisting of 10000 frames sampled without replacement from each block based on the metadynamics weights.

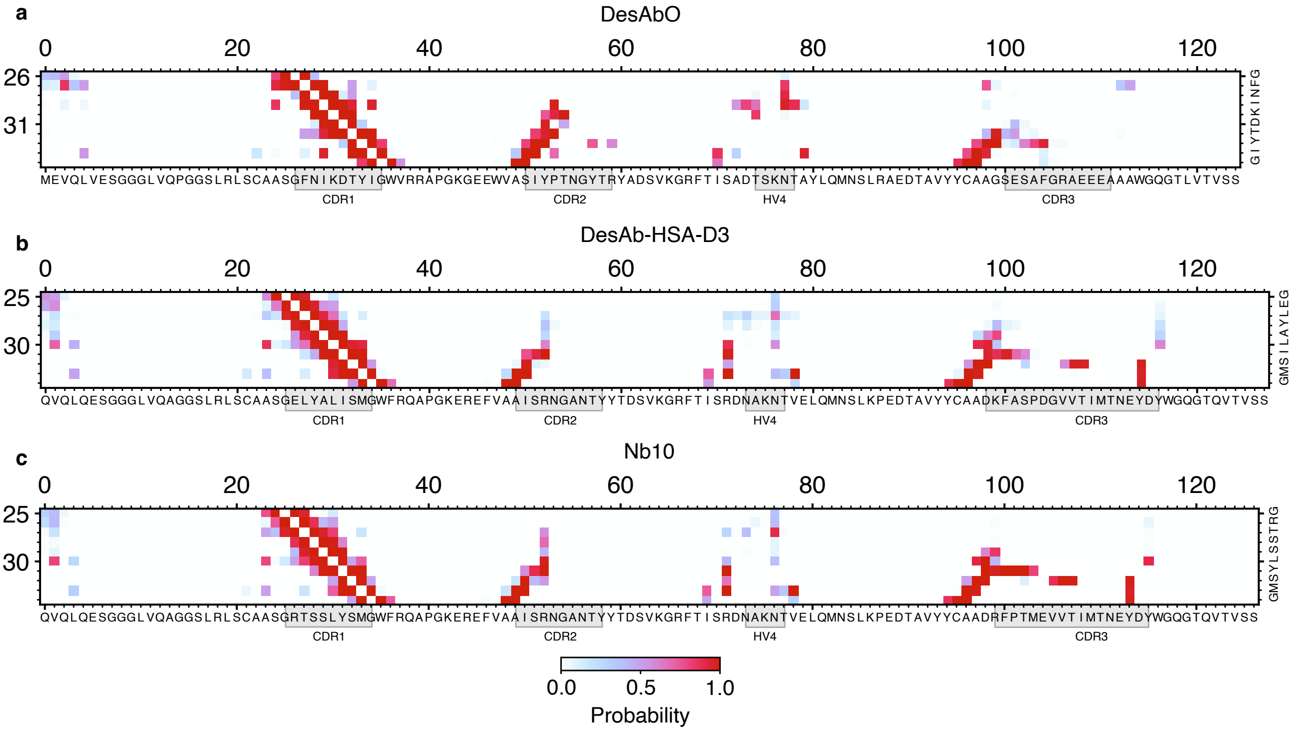

Figure S5. Comparison of CDR1-scaffold contacts for DesAbO (**a**), DesAb-HSA-D3 **b** and Nb10 (**c**).

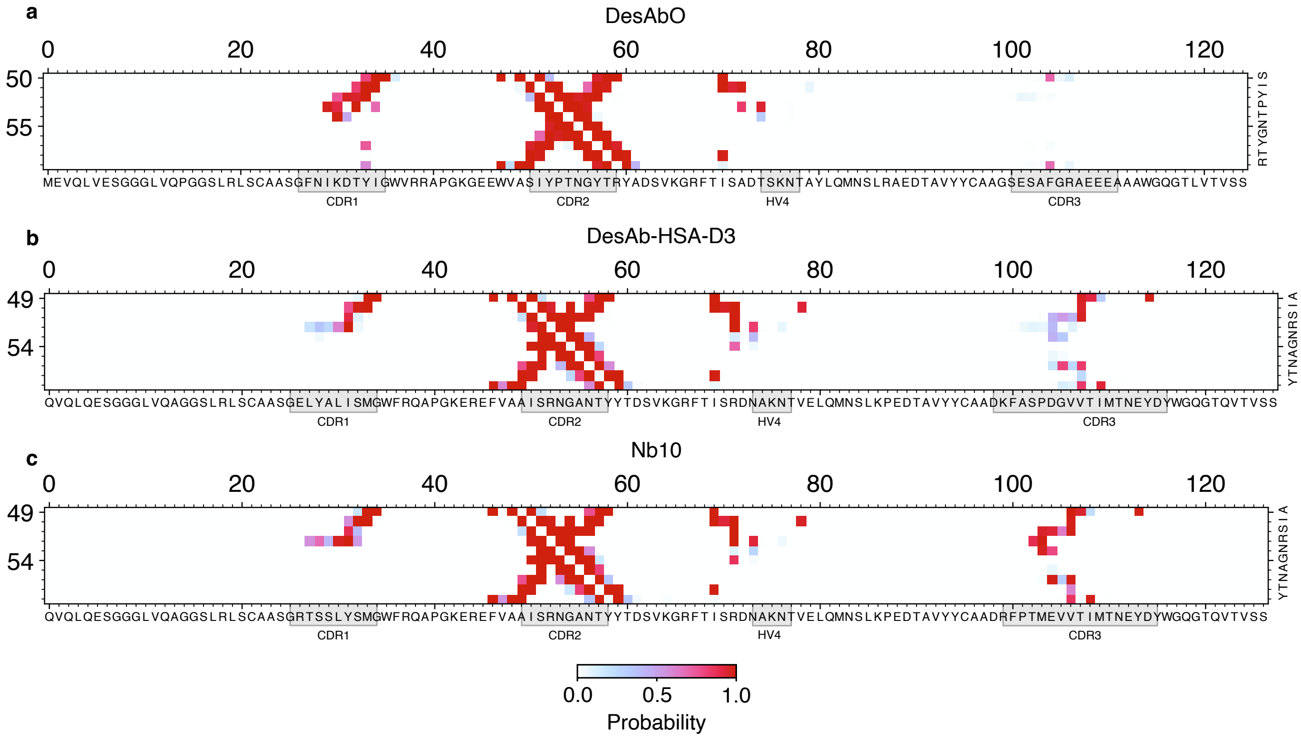

Figure S6. Comparison of CDR2-scaffold contacts for DesAbO (**a**), DesAb-HSA-D3 **b** and Nb10 (**c**).

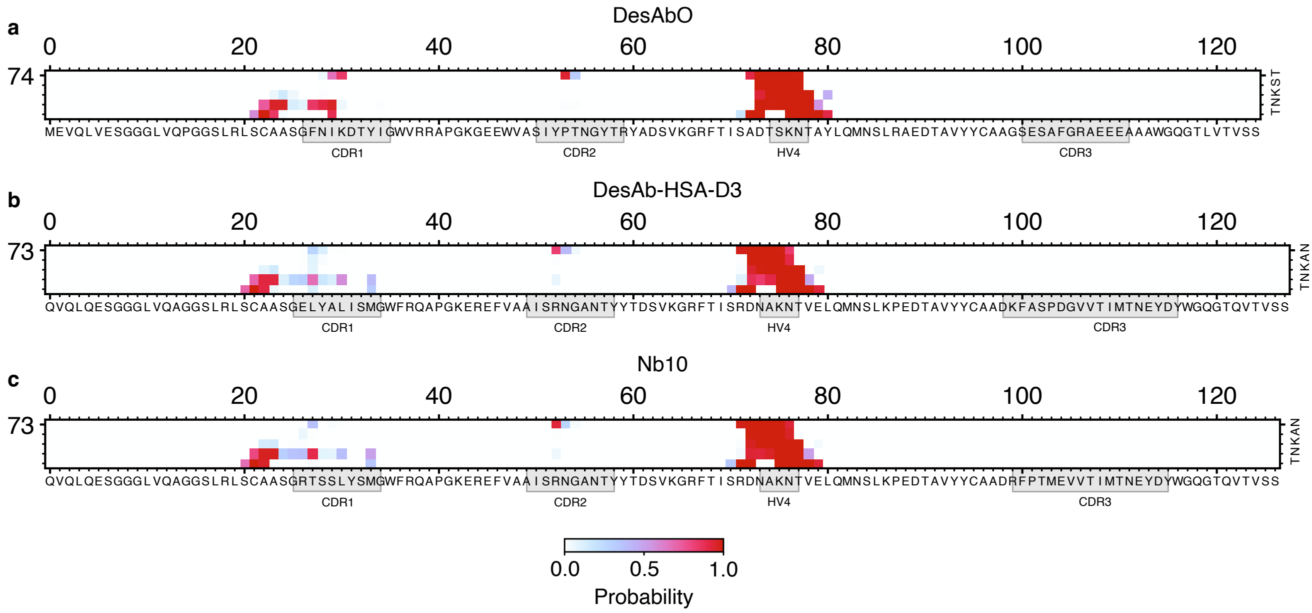

Figure S7. Comparison of HV4-scaffold contacts for DesAbO (**a**), DesAb-HSA-D3 **b** and Nb10 (**c**).

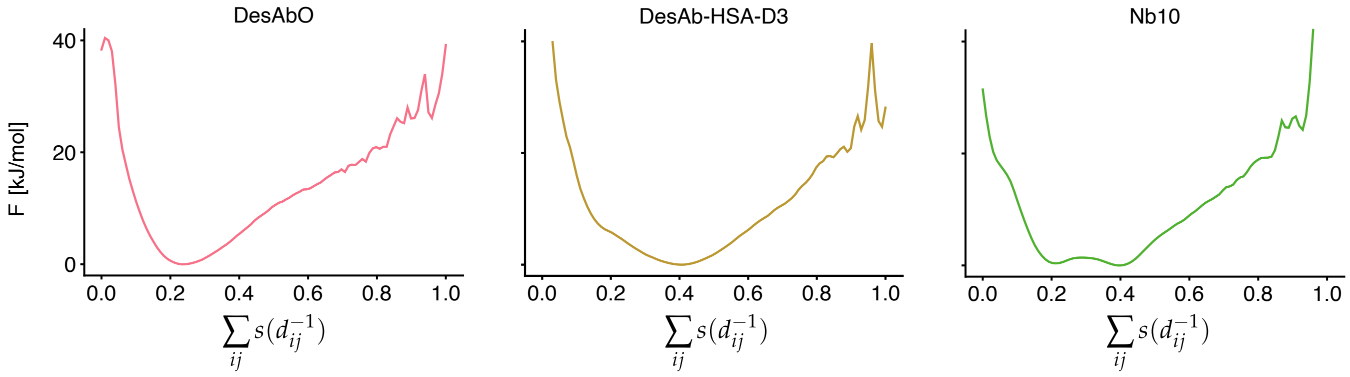

Figure S8. Comparison of the free energy surfaces for the HV4 loop region. The chosen coordinate represents the sum of inverse minimum distances between the loop region and the rest of the sdAb, modulated by a switching function $s(x) = \left(1 - \frac{x}{r_0}\right)^6 \left(1 - \frac{x}{r_0}\right)^{-12}$ with a cutoff of $r_0 = 0.45$ nm and normalised to span from 0 to 1. This procedure was chosen over contacts to avoid discontinuities resulting from the low number of residues in the loop.

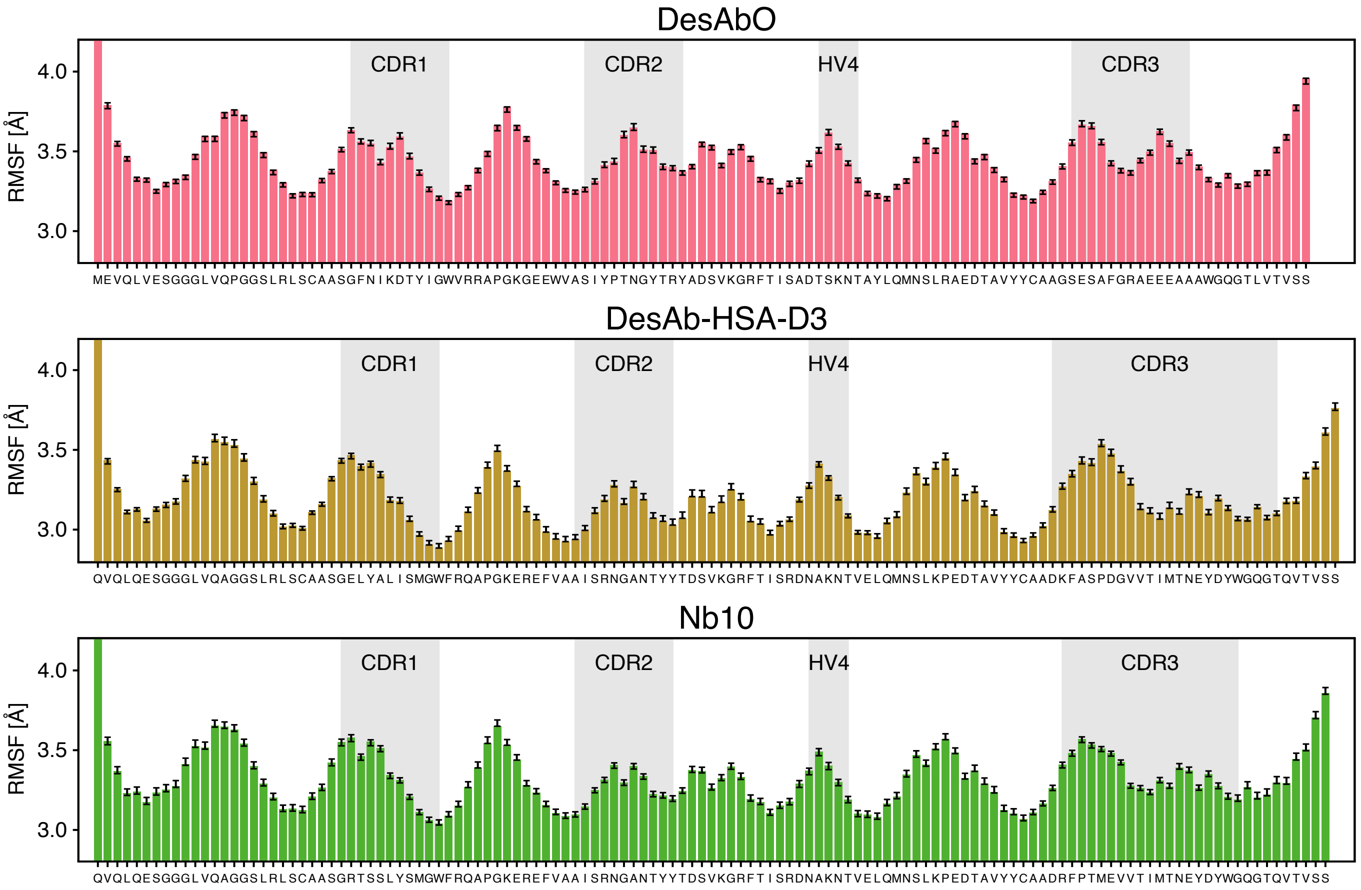

Figure S9. Root-mean-square fluctuation of each residue with respect to the center of the most populated cluster. Error bars indicate the 95th percentile of the bootstrap sample-of-the-mean over all 20 samples consisting of 10000 frames sampled from each block based on the metadynamics weights.
